# Supplementary material for: Altered microRNA Transcriptome in Cultured Human Liver Cells upon Infection with Ebola Virus
Source: Int J Mol Sci. 2021 Apr 6;22(7):3792. doi: 10.3390/ijms22073792 (PMC8038836; doi:10.3390/ijms22073792)
Supplement: Supplementary file 1 [file ijms-22-03792-s001.zip › Supplementary_File/D_ KEGG_Analysis_Results/16-30nt_pathway_Mayinga-96h-Huh7_vs_Control-96h-Huh7_up.mature_mirna_targets/hsa_pathwayResult.html]

| PathwayID | Definition | OriginalWebSite | Fisher-Pvalue | SelectionCounts | SelectionSize | Count | Size | FDR | Enrichment\_Score | GeneRatio | Genes |
| --- | --- | --- | --- | --- | --- | --- | --- | --- | --- | --- | --- |
| hsa03013 | RNA transport - Homo sapiens (human) | http://www.genome.jp/kegg-bin/show\_pathway?hsa03013+1964+8661+1977+1983+22916+51068+132430+10775+7329+65110 | 3.550834e-03 | 10 | 175 | 165 | 7906 | 5.969704e-01 | 2.449670 | 0.057143 | EIF1AX//EIF3A//EIF4E//EIF5//NCBP2//NMD3//PABPC4L//POP4//UBE2I//UPF3A |
| hsa04392 | Hippo signaling pathway - multiple species - Homo sapiens (human) | http://www.genome.jp/kegg-bin/show\_pathway?hsa04392+92597+4771+9770+83937 | 3.574673e-03 | 4 | 175 | 29 | 7906 | 5.969704e-01 | 2.446764 | 0.022857 | MOB1B//NF2//RASSF2//RASSF4 |
| hsa05330 | Allograft rejection - Homo sapiens (human) | http://www.genome.jp/kegg-bin/show\_pathway?hsa05330+959+355+3115+3558 | 9.523267e-03 | 4 | 175 | 38 | 7906 | 8.471137e-01 | 2.021214 | 0.022857 | CD40LG//FAS//HLA-DPB1//IL2 |
| hsa00561 | Glycerolipid metabolism - Homo sapiens (human) | http://www.genome.jp/kegg-bin/show\_pathway?hsa00561+56895+55326+219+1606+346606 | 1.098748e-02 | 5 | 175 | 61 | 7906 | 8.471137e-01 | 1.959102 | 0.028571 | AGPAT4//AGPAT5//ALDH1B1//DGKA//MOGAT3 |
| hsa04940 | Type I diabetes mellitus - Homo sapiens (human) | http://www.genome.jp/kegg-bin/show\_pathway?hsa04940+355+3115+3558+4049 | 1.462224e-02 | 4 | 175 | 43 | 7906 | 8.471137e-01 | 1.834986 | 0.022857 | FAS//HLA-DPB1//IL2//LTA |
| hsa04145 | Phagosome - Homo sapiens (human) | http://www.genome.jp/kegg-bin/show\_pathway?hsa04145+8992+30835+10332+1783+3115+7037+7057+347733 | 1.939577e-02 | 8 | 175 | 152 | 7906 | 8.471137e-01 | 1.712293 | 0.045714 | ATP6V0E1//CD209//CLEC4M//DYNC1LI2//HLA-DPB1//TFRC//THBS1//TUBB2B |
| hsa00280 | Valine, leucine and isoleucine degradation - Homo sapiens (human) | http://www.genome.jp/kegg-bin/show\_pathway?hsa00280+27034+219+64087+5019 | 2.118009e-02 | 4 | 175 | 48 | 7906 | 8.471137e-01 | 1.674072 | 0.022857 | ACAD8//ALDH1B1//MCCC2//OXCT1 |
| hsa04070 | Phosphatidylinositol signaling system - Homo sapiens (human) | http://www.genome.jp/kegg-bin/show\_pathway?hsa04070+1606+3633+4534+8394+23396+8867 | 2.223779e-02 | 6 | 175 | 99 | 7906 | 8.471137e-01 | 1.652908 | 0.034286 | DGKA//INPP5B//MTM1//PIP5K1A//PIP5K1C//SYNJ1 |
| hsa00562 | Inositol phosphate metabolism - Homo sapiens (human) | http://www.genome.jp/kegg-bin/show\_pathway?hsa00562+3633+4534+8394+23396+8867 | 2.368391e-02 | 5 | 175 | 74 | 7906 | 8.471137e-01 | 1.625547 | 0.028571 | INPP5B//MTM1//PIP5K1A//PIP5K1C//SYNJ1 |
| hsa00030 | Pentose phosphate pathway - Homo sapiens (human) | http://www.genome.jp/kegg-bin/show\_pathway?hsa00030+229+9563+6120 | 2.793271e-02 | 3 | 175 | 30 | 7906 | 8.471137e-01 | 1.553887 | 0.017143 | ALDOB//H6PD//RPE |
| hsa05320 | Autoimmune thyroid disease - Homo sapiens (human) | http://www.genome.jp/kegg-bin/show\_pathway?hsa05320+959+355+3115+3558 | 2.929216e-02 | 4 | 175 | 53 | 7906 | 8.471137e-01 | 1.533249 | 0.022857 | CD40LG//FAS//HLA-DPB1//IL2 |
| hsa04710 | Circadian rhythm - Homo sapiens (human) | http://www.genome.jp/kegg-bin/show\_pathway?hsa04710+23291+5563+6095 | 3.043522e-02 | 3 | 175 | 31 | 7906 | 8.471137e-01 | 1.516623 | 0.017143 | FBXW11//PRKAA2//RORA |
